# Membership Function-Weighted Non-Linear Fitting Method for Optical-Sensing Modeling and Reconstruction

**DOI:** 10.3390/s18113762

**Published:** 2018-11-03

**Authors:** Shuo Meng, Zhenhui Du, Liming Yuan, Shuanke Wang, Ruiyan Han, Xiaoyu Wang

**Affiliations:** State Key Laboratory of Precision Measuring Technology and Instruments, Tianjin University, Tianjin 300072, China; mengsh@tju.edu.cn (S.M.); yuanliming@tju.edu.cn (L.Y.); wangshuanke@tju.edu.cn (S.W.); hanruiyan@tju.edu.cn (R.H.); wangxiaoyu9453@tju.edu.cn (X.W.)

**Keywords:** optical-sensing signal processing, non-linear curve fitting, laser-absorption spectroscopy, image reconstruction-restoration, sensing modeling and reconstruction

## Abstract

Imprecise measurements present universally due to variability in the measurement error. We devised a very simple membership function to evaluate fuzzily the quality of optical sensing with a small dataset, where a normal distribution cannot be assumed. The proposed membership function was further used as a weighting function for non-linear curve fitting under expected mathematical model constraints, namely the membership function-weighted Levenberg–Marquardt (MFW-LM) algorithm. The robustness and effectiveness of the MFW-LM algorithm were demonstrated by an optical-sensing simulation and two practical applications. (1) In laser-absorption spectroscopy, molecular spectral line modeling was greatly improved by the method. The measurement uncertainty of temperature and pressure were reduced dramatically, by 53.3% and 43.5%, respectively, compared with the original method. (2) In imaging, a laser beam-profile reconstruction from heavy distorted observations was improved by the method. As the dynamic range of the infrared camera increased from 256 to 415, the detailed resolution of the laser-beam profiles increased by an amazing 360%, achieving high dynamic-range imaging to capture optical signal details. Therefore, the MFW-LM algorithm provides a robust and effective tool for fitting a proper physical model and precision parameters from low-quality data.

## 1. Introduction

Curve fitting is one of the most powerful and most widely used analysis tools to pre- and post-process data [1,2], to remove outliers [3,4], to compare candidate models [5], and to examine the relationship between one or more predictors [6,7,8]. The parameterization of curve fitting is usually realized by minimizing linear or non-linear least-squares residuals [9,10], in which the Levenberg–Marquardt (LM) algorithm, also known as the damped least-squares method, is popular for fast convergence [11]. Whether the LM fitting results can reflect physical meaning is highly dependent on the quality of measured signal. Improving the LM algorithm’s robustness from outliers and reducing its dependence on the quality of the measured signal are desired.

The LM algorithm for curve fitting was first proposed by Levenberg [12] in 1943 and optimized by Marquardt [13] in 1963. The idea of weighted fitting was demonstrated in 1959. Asadi et al. [14] proposed a hybrid intelligent model combination of genetic algorithms and the LM algorithm for stock-exchange index prediction to cope with the fluctuations of stock-market values. Nawi et al. [15] proposed an algorithm in which a cuckoo search algorithm was used to provide optimal weights for the LM algorithm to obtain better results in training feed-forward artificial neural networks. Zhang et al. [16] proposed a finite-element analysis-based LM algorithm to extract the features of the Brillouin-scattering spectrum with different linewidths and signal-to-noise ratios (SNRs). The weighted LM algorithm had attracted much attention for potentially reducing the effect of outliers.

Outliers present universally due to variability in measurement error, including a transient malfunction of apparatuses, errors in data transmission or transcription, changes in system behavior, operation error, instrument error or through natural deviations, and a flaw in the assumed theory. These outliers always lower the goodness of fit and the precision of fitted parameters. There is no rigid mathematical definition of what constitutes an outlier; determining whether or not an observation is an outlier is ultimately a subjective exercise. Outlier detection methods, e.g., Chauvenet’s criterion [17], Grubbs’ test [18], Dixon’s Q test [19], and Peirce’s criterion [20], are usually graphically or model-based. Deletion of outliers is a controversial practice frowned upon by scientists because the deletion process does not make the practice more scientifically or methodologically sound, especially in small sets or where a normal distribution cannot be assumed. Furthermore, when non-linear fitting is performed from small numbers of replicated observations, the results of an analysis can be very badly and unpredictably affected.

Optical sensing is often performed in situ or in online applications, where small datasets are common for the variability of working conditions or fast response time. These results in outliers buried in observations being more difficult to distinguish from low-quality data distorted by non-linear transmission, interference, electrical noise, dark current fluctuation (DCF), and dark-current non-uniformity and photoresponse non-uniformity between pixels for optical image sensors [21,22,23]. Outliers and distorted signal can be both overly influenced the accuracy of in situ optical sensing and modeling. There is not any universal or robust algorithm to improve the goodness of fit and the precision of fitted parameters for outliers-contained optical sensing with small datasets.

In the work described in this paper, we considered the outliers and distorted signal of optical sensing together as imprecise measurements. Taking the idea of fuzzy logic for the first time, we devised a membership function for optical-sensing data to evaluate the observations’ fidelity. Furthermore, we used the membership function as the weighting function of fitting for signal modeling and reconstruction under expected mathematical model constraints, i.e., a membership-function-weighted Levenberg–Marquardt (MFW-LM) algorithm. We demonstrated the MFW-LM algorithm by an optical-sensing simulation and two experiments: (1) laser-absorption-spectroscopy analysis and (2) laser-beam-profile measurement.

## 2. Method

### 2.1. Optical-Sensing Membership Function

We considered the outliers and distorted signal of optical sensing together as imprecise measurements, which present universally due to three phenomena: (1) imperfect response of sensing and signal conditioning due to non-linearity, DCF, and performance decline with device aging; (2) interference from stray light and optical fringe [24,25], power-line interference [26], and electromagnetic interference; and (3) electrical noise from electronic devices with various processes, including thermal noise, shot noise, flicker noise, burst noise, and transit-time noise.

**Figure 1:**
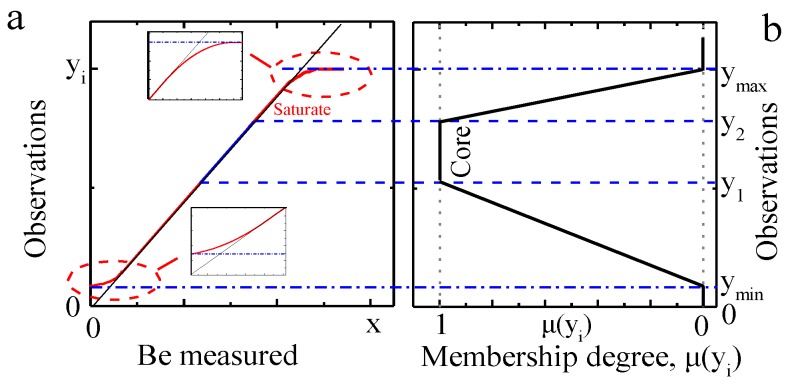
(**a**) Typical responsivity of a photosensor; (**b**) assignment of a membership function. *y*_max_, *y*_min_, *y*_1_ and *y*_2_ are the maximum, minimum, and two critical observations, respectively. The two small panels in (a) are partial enlargements at the responsivity’s ends.

Generally, the measurement reliability of optical sensing depends largely on the responsivity of the adopted photosensor and signal conditioning unit. A typical responsivity of a photosensor usually exhibits quite good linearity in the middle region of its response curve, while presenting non-linearity near the two ends of the response curve, as shown in Figure 1a. A slightly different responsivity at the low-input end and a saturated feature at the up-input end are very common for most photosensors. The signal conditioning unit of an optical system is usually used to perform amplification for high resolution and SNR of the input signal, filtering for minor out-band interference, and electrical isolation for isolating possible sources of signal perturbations. Practically, a variety of interference and noise should be reduced to a rather small amount compared with the measured signal.

Therefore, the reliability of measured data is closely related to the signal magnitude for a well-designed optical system. Random noise is statistical noise having a Gaussian distribution. Thus, the measurements corresponding to the middle and upper parts of the response curve are more reliable than those of the two ends of the curve.

We took the idea of fuzzy logic, where a membership function represents the degree of truth as an extension of valuation [27]. For an optical-sensing dataset [*y_i_*] (*i* = 1,2, …, *n*), we devised a membership function, denoted *μ*(*y_i_*), mapping from [*y_i_*] to the real unit interval [0, 1], for optical-sensing datum *y_i_* (*i* = 1, 2, …, *n*) to quantify its fidelity:

(1) $$\mu (y_{i})=\{\begin{array}{c} 0\\ \left(1/y_{1}\right)\times y_{i}\\ 1\\ \left(y_{\max}-y_{i})/(y_{\max}-y_{2}\right) \end{array}\begin{array}{c} y_{i}\leq y_{\min},y_{i}\geq y_{\max}\\ y_{\min}<y_{i}<y_{1}\\ y_{1}\leq y_{i}\leq y_{2}\\ y_{2}<y_{i}<y_{\max} \end{array}\;$$

(2) $$\begin{array}{cc} \mu (y_{i})\in [0,1] & i=1,2,\cdots ,n \end{array}\;$$

where *μ*(*y_i_*) is the membership degree of an observation *y_i_*; *y*_max_ and *y*_min_ are the maximum and the minimum of right observation, respectively; *y*_1_ and *y*_2_ are two critical observations with high confidence, respectively; and n is the number of observations.

The criteria for the key parameters in the membership function are closely related to system characteristics. The *y*_max_ and *y*_min_ are limited values of a sensor’s output range. Any observations beyond *y*_max_ and *y*_min_ must be outliers, and we assigned membership degree 0 to them, i.e., their fidelity is none. *y*_1_ and *y*_2_ have fuzzy certainty, which is used to determine the range with the best linearity and make fuzzy optimal selection of observations. These observations between the two critical points *y*_1_ and *y*_2_ have the highest confidence, and we assigned membership degree 1 to them, which comprise the core of the membership function. However, the scope of this area is not strictly defined and can be appropriately adjusted according to different sensor responsivity and experimental purposes. We assigned membership degree 0 to 1 for the other observations based on their measurement value. The functions from *y*_min_ to *y*_1_, and from *y*_2_ to *y*_max_ are specifically designed on the basis of the respective major problems to be solved. The signal in the low amplitude interval is greatly affected by the random noise, so it is necessary to reduce the contribution of the low-SNR observations; while the signal of the high amplitude interval mainly addresses the difficulties of signal distortion and saturation caused by the non-linearity of the sensor’s responsivity. A membership function assigned for a typical responsivity of a photosensor (Figure 1a) is shown in Figure 1b.

The membership degree *μ*(*y_i_*) is closely related with the magnitude of the measurement, similar to the experience using an analog instrument, i.e., one always achieves better accuracy at approximately two-thirds of the full scale of an instrument by properly selecting its scale range [28]. Therefore, the interval set by the two critical values *y*_1_ and *y*_2_ of the membership function is usually chosen to be near two-thirds of the observations or sensor’s responsivity. A higher membership degree *μ*(*y_i_*) means a precise measurement, while a smaller *μ*(*y_i_*) implies the poorer fidelity of observations. The membership function, drawing on the ideology of fuzzy logic, is a novel concept and valuable for quantitative simple evaluation of observations under the premise that the SNR of the observations is within a reliable range. The higher the SNR of the observations, the more objective the fitting result of the MFW-LM algorithm will be.

### 2.2. Membership Function-Weighted Levenberg–Marquardt (MFW-LM) Algorithm

The LM algorithm is a well-known damped least-squares algorithm [29], so only a brief review is given. For an observed data set [*y_i_*] (*i* = 1,2, …, *n*), the expected mathematical model f(*x*, $\vec{a}$) is often determined based on previous knowledge. The parameter vector $\vec{a}$ of the model is approximated within a trustworthy range (denoted k, where |k| < ε and ε is any positive number) near its initial value $\vec{a_{k}}$ to seek an optimal parameter vector $\vec{a_{f}}$ that minimizes the sum of squared residuals *R*($\vec{a}$).

The fitting criteria of the LM algorithm is given by [30,31]:

(3) $$\min R(\vec{a})=\sum_{i=1}^{n}{\left[y_{i}-y_{i}(\vec{a_{{}^{k}}}+k)\right]}^{2},i=1,2,\ldots ,n\;$$

where *R*($\vec{a}$) is the sum of the squared residuals, *y_i_*($\vec{a_{k}}$ + k) is the data when the parameter is estimated as ($\vec{a_{k}}$ + k), and n is the number of fitted points.

We promoted the fitting quality of optical sensing by using the aforementioned membership function as a weighting function. The fitting criterion in Equation (3) was weighted by the membership degree of observations described by Equation (1), namely the MFW-LM algorithm, which is described as follows:

(4) $$\min R(\vec{a})=\sum_{i=1}^{n}\mu \left(y_{i}\right)[y_{i}-y_{i}(\vec{a_{{}^{k}}}+k){]}^{2},i=1,2,\ldots ,n\;$$

where *μ*(*y_i_*) is a membership degree used as a regression weight; $\vec{a_{k}}$ is the parameter vector of the expected mathematical model. We substituted the optimal parameter vector $\vec{a_{f}}$ that meets the improved fitting criteria [Equation (4)] into f(*x*, $\vec{a}$) to obtain the fitting result that is in better agreement with the physical meaning.

**Figure 2:**
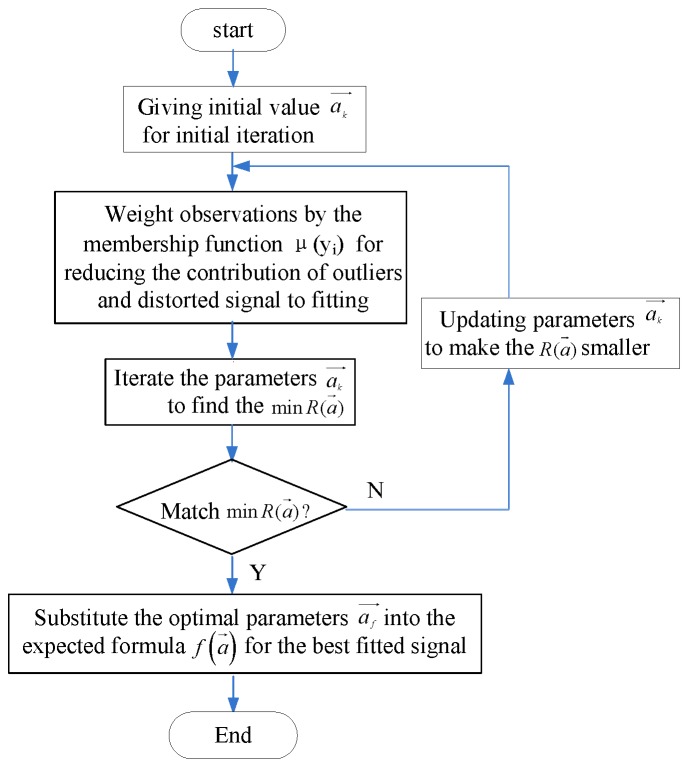
Flowchart of membership function-weighted Levenberg–Marquardt (MFW-LM) fitting process.

We programmed the entire algorithm in OriginPro and LabView software. First, we predicted the initial values of parameters by the least-squares method or experimental results. Next, we weighted observations by assigning a membership function. The fitting parameters were continuously involved in the iterative process until the sum of squared residuals was minimized. Finally, the optimal parameters were obtained to complete the MFW-LM algorithm. The flowchart of this process is shown in Figure 2.

From the view of weighted curve fitting, the membership function is a rather simple function capable of coping with imprecise measurements.

## 3. Verification of MFW-LM Algorithm

We verified the MFW-LM algorithm by simulating optical-sensing signals, which were accompanied by random noise, sensor non-linearity, and data distortion. We compared the fitting results of the proposed MFW-LM algorithm with that of an outliers-excluded LM (OE-LM).

**Figure 3:**
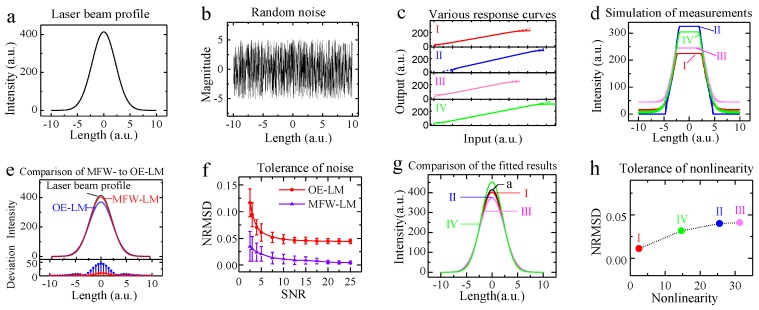
Demonstration of MFW-LM algorithm. (**a**) Simulated laser-beam profile with Gaussian distribution; (**b**) random noise; (**c**) illustration of various response curves; (**d**) simulated signals corresponding to various response curves in c and random noise in b; (**e**) comparison between original (a) and fitted values by the MFW- and outliers-excluded (OE)-LM algorithms with response curve I in c; (**f**) noise-tolerance comparison of MFW- and OE-LM algorithms with response curve I in c; (**g**) comparison of fitted results with various measurements in d; (**h**) non-linearity tolerance of MFW-LM algorithm. MFW-LM denotes membership function-weighted Levenberg–Marquardt algorithm; OE-LM denotes outliers-excluded Levenberg-Marquardt algorithm; NRMSD denotes normalized root-mean-square deviation.

We assumed a laser beam featuring a Gaussian function based on a large number of previous studies [32,33,34,35], which was described by Equation (5) and is shown in Figure 3a:

(5) $$I=I_{0}+\left(\eta /(w\times \sqrt{\pi /2})\right)\times e^{-2{\left(\left(x-x_{c}\right)/w\right)}^{2}}\;$$

where *I*_0_ can be seen as background light; *η* is a constant; *w* is the radius at which the intensity value falls to 1/*e*^2^ of the maximum intensity; *x_c_* is the offset of the maximum intensity relative to the horizontal axis; *x* is the radial distance from the center axis of the beam; all parameters were randomly generated (in Table 1).

The laser beam was measured by image sensors with four different response characteristics as shown in Figure 3c and with random noise shown in Figure 3b. Taking the simulated SNR = 25, calculated by the amplitude of the simulated signal and the random noise, as an example, we simulated the measurements. The measured laser beams with four image sensors are shown in Figure 3d, in which we intended a bigger input to bring about a saturated signal, seriously distorted data, or outliers, to verify the proposed MFW-LM algorithm.

We fitted measurement I (in Figure 3d) with the MFW- and OE-LM algorithms separately. The fitted curves and preset Gaussian function are shown in the upper panel of Figure 3e and their deviation in the lower panel of Figure 3e. We then changed the response of image sensors to curves II–IV (in Figure 3c), which is more non-linear owing to device aging, and fitted the curves I–IV in Figure 3c with MFW-LM algorithm to verify its universality, as shown in Figure 3g. We simulated 100 signals at different SNRs by adjusting the amplitude of random noise and the sensor’s non-linearity, and averaged them to obtain the relative normalized root-mean-square deviation (NRMSD), described in Equation (6), of the fitted and preset values, as shown in Figure 3f,h, respectively.

According to measurements I–IV, we set the parameters of the membership function (in Table 2) for MFW-LM fitting based on the response curves I–IV in Figure 3c. The *y*_max_ and *y*_min_ are limited values of the sensor’s responsivity. *y*_1_ and *y*_2_ are the two-thirds of the sensor’s responsivity. The parameter vector $\vec{a}$ = (*I*_0_, *η*, *w*, *x_c_*) was obtained by LM fitting and was used as the initial parameter vector of the MFW-LM algorithm. After iteration, the final iteration obtained the optimal parameter vector, which can minimize the sum of squared residuals *R*($\vec{a}$). Substituting it into the Gaussian function yielded the fitted laser beam profile.

We compared the deviation between the reconstructed signal and the preset laser-beam profile, which was quantified by the NRMSD of the relative residuals as follows:

(6) $$\mathrm{NRMSD}=\sqrt{\sum_{i=1}^{N}{\left[\left(y_{i}^{\prime }-y_{i}^{*}\right)/y_{\max}^{\prime }\right]}^{2}/n}$$

where $y_{i}^{\prime }$ and $y_{i}^{*}$ are the fitted data and the preset value, respectively; $y_{\max}^{\prime }$ is the maximum of $y_{i}^{\prime }$; and *n* is the number of fitted points.

The sensor’s non-linearity *δ* was expressed by Equation (7) and demonstrated in Figure 1a:

(7) $$\delta =\sum \left|y_{n}-y_{l}\right|/Y\;$$

where *y_n_* is the non-linear response data, *y*_l_ the linear response data, and *Y* the range of linear response.

The result of the single simulation (Figure 3e) indicates that the relative NRMSD of the OE-LM and the MFW-LM algorithms were 4.5% and 1.1%, respectively. Multiple simulations at different SNRs found that the NRMSD of the MFW-LM algorithm was improved by 66.0%–89.7% compared to the OE-LM algorithm, and that it is always within 5% for different response characteristics when the SNR is not less than 2.5 or the sensor’s non-linearity is not greater than 31.32.

Therefore, the MFW-LM algorithm is extremely robust and effective in minimizing the contribution of outliers and distorted signals and accurately reconstructs the laser-beam profile when dealing with optical sensing with small datasets; that is, the proposed MFW-LM algorithm is suitable for curve fitting that requires high-precision measurement.

## 4. Applications

### 4.1. Optical Absorption Spectroscopy Analysis

To improve the accuracy of optical absorption spectroscopy analysis, which is often frustrated by imperfect response, interference, and electronic noise [24,25,36,37,38] in signal acquisition and processing, we applied the MFW-LM algorithm to molecular absorption line modeling of H_2_O and compared the measurement uncertainty of the temperature and pressure calculated by the fitting results of the MFW-LM and the LM algorithms.

H_2_O spectral intensity and line shape were used to calculate temperature, and pressure by the LM algorithm based on wavelength-modulation spectroscopy (WMS) [39]. The fitted mathematical expression was determined by the center absorption wavenumber *ν*_0_, integral absorbance *A_i_*, and Lorentzian broadening Δ*_νL_* [40]. The molecular absorption line modeling *M* that was used for the fitting is introduced by Equations (8)–(10):

(8) $$M=\frac{A}{i_{0}\cdot \cos\psi }\left[H_{2}+\frac{i_{0}}{2}\left(H_{1}+H_{3}\right)\cos\psi \right],\;$$

(9) $$H_{i}=-\frac{1}{\pi }\int_{-\pi }^{\pi }\varphi_{v}\left(\bar{\nu }+a\cos\theta ,\Delta_{\nu_{L}},\Delta_{\nu_{G}}\right)\cos i\theta d\theta ,\;$$

(10) $$\varphi_{v}\left(\nu -\nu_{0},\Delta \nu_{v}\right)\approx c_{L}\frac{1}{\pi }\frac{\Delta \nu_{v}}{{\left(\nu -\nu_{0}\right)}^{2}+\Delta \nu_{v}}+c_{G}\frac{\sqrt{\ln2}}{\sqrt{\pi }\Delta \nu_{v}}\exp\left[-\ln2{\left(\frac{\nu -\nu_{0}}{\Delta \nu_{v}}\right)}^{2}\right],\;$$

where *M* is the molecular absorption line modeling; *A* is integral absorbance; *i*_0_ = 0.036 and *ψ* = 1.12π are parameters of the laser; *H_i_* is the *i*th harmonic signal; *φ_v_* is the Voigt profile; $\bar{\nu }=2.0\times 10^{5}$ GHz is the center frequency of the laser; *a* = 0.065 cm^−1^ is the modulation amplitude; *θ*∈[−π, π]; *ν* is absorption wavenumber; ν_0_ is the center absorption wavenumber; Δ*_νL_* is the Lorentzian broadening; Δ*_νG_* is the Gaussian broadening; $\Delta \nu_{v}$ is the half width at half maximum (HWHM) of Voigt; *c_L_* and *c_G_* are weights, which are related to the absorption lineshape [41].

Gas temperature T was calculated according to the line-strength ratio R = *A_i_*/*A_j_* [Equation (11)], which is a fourth-order polynomial obtained by fitting the data provided by the HITRAN database [42]. Pressure P can be inferred by combining Equations (12) to (13):

(11) $$T=g(R)=k_{1}+k_{2}\times R+k_{3}\times R^{2}+k_{4}\times R^{3}+k_{5}\times R^{4}\;$$

(12) $$\Delta_{\nu_{L}}=P\cdot \left[\chi_{H_{2}O}\cdot \gamma_{\mathrm{self}}+\left(1-\chi_{H_{2}O}\right)\cdot \gamma_{\mathrm{mix}}\right],\;$$

(13) $$\chi_{H_{2}O}=\frac{A}{P\cdot S\cdot L},\;$$

where *T* is the gas temperature; *k*_1_, *k*_2_, *k*_3_, *k*_4_, and *k*_5_ are polynomial coefficients (Table 3); *R* is the line-strength ratio; Δ*_νL_* is Lorentzian broadening; $\chi_{H_{2}O}$ is the concentration of H_2_O; *γ*_self_ and *γ*_mix_, calculated by $\gamma_{j}\left(T\right)=\gamma_{j}\left(T_{0}\right){\left(T_{0}/T\right)}^{n_{j}}$, are the self-broadening coefficient of H_2_O and the mix-broadening coefficient of H_2_O with other gas compounds, respectively; $\gamma_{j}\left(T_{0}\right)$ is the broadening factor at the reference temperature; *n_j_* is the temperature index, which is typically 0.5; A is integral absorbance; *S* is the line strength; and *L* is the optical path length.

The temperature of the experimental environment measured by a thermocouple was T = 350 K. Then, the pressure calculated according to the ideal gas law was P = 1.18 atm. The temperature and pressure measurement uncertainties obtained by the LM algorithm were 2.6% and 6.8%, respectively.

To weaken the roles of outliers and distorted signals on curve fitting, we used the proposed MFW-LM algorithm to refit the raw observations. The empirical estimates of *ν*_0_, *A_i_* and Δ*_νL_* were regarded as initial parameters. The parameters and weighting functions (the membership functions) were set for the LM fitting program by Labview software. By repeating multiple iterations, once the sum of squared residuals *R*($\vec{a}$) minimizing, the fitting process will converge to the unique solution, the optimal parameters, and the molecular absorption lineshape of H_2_O was obtained by the MFW-LM fit. Based on the robustness of the MFW-LM algorithm, the observed signals obtained by sensors with different responsivity can be fitted. Observations and sensor responsivity are one-to-one corresponding, so the limit values and critical values of the observations can be taken as the key parameters of the membership function (see Table 4), so that the responsivity of the photodetector (PDA10CS-EC, Thorlabs Inc., Newton, NJ, USA) used in the experiment is not needed to be tested or corrected. The parameters of the membership function, following Equation (1), were set based on the molecular absorption lineshape of H_2_O collected by this photodetector as shown in Table 4. The *y*_max_ and *y*_min_ are limited values of the observations’ amplitude. *y*_1_ and *y*_2_ are the two thirds of the observations’ amplitude. The variables of the molecular absorption line modeling of H_2_O are continually updated and iterated until a set of variables is found to minimize the *R*($\vec{a}$) (Equation (4)).

**Figure 4:**
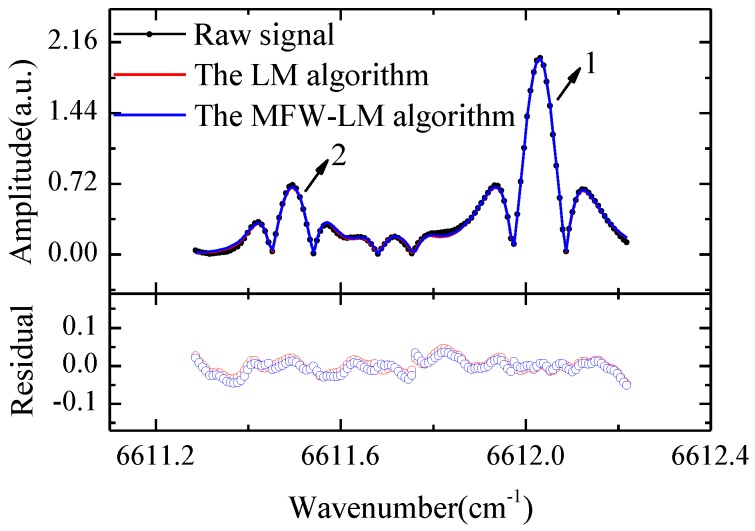
The raw molecular absorption lineshape of H_2_O and the fitting results of LM and MFW-LM algorithms. The two absorption peaks 1, 2 analyzed are shown in the figure.

The molecular absorption lineshape of H_2_O and the fitting results of the LM and the MFW-LM algorithms are shown in Figure 4 and Table 5.

The integral absorbance ratio (i.e., R) was obtained from the two absorption peaks 1 and 2 of H_2_O. Substituting it into Equation (11), we can calculate the gas temperature T. In Equations (12) and (13), the parameters other than the pressure P and H_2_O concentration $\chi_{H_{2}O}$ are constants or the known parameters obtained by the fitting signal. Therefore, the pressure P and H_2_O concentration $\chi_{H_{2}O}$ can be obtained by combining Equations (12) and (13). The calculated results and uncertainty analysis of the gas temperature T and pressure P are shown in Table 5.

**Figure 5:**
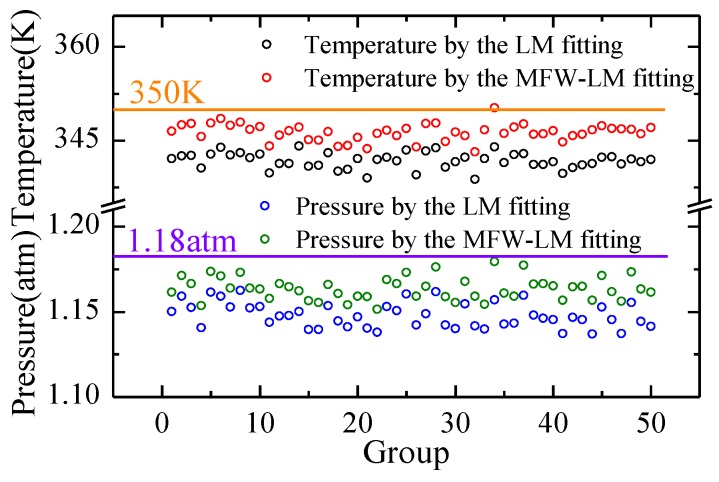
Comparison of the key parameters obtained by LM and MFW-LM algorithms with truth values in 50 repeated experiments. Orange and purple horizontal lines are truth values of gas temperature (350 K) and pressure (1.18 atm), respectively.

We then took the measurement results from 50 repeated experiments under the same experimental conditions to compare them with the truth values, as shown in Figure 5.

The relative standard deviation (RSD) was used to represent the calculation error of key parameters:

(14) $$\mathrm{RSD}=\frac{\sqrt{\sum_{i=1}^{n}(v_{i}-v_{b}{)}^{2}}}{v_{b}\sqrt{n-1}}\times 100\%\;$$

where *v_i_* is the calculated value, *v_b_* the truth value, and *N* the number of data points.

It can be seen that while the MFW-LM algorithm resulted in a slightly lower NRMSD of the signal line profile, it reduced the relative error of the gas temperature and pressure calculated from the fitting result by 42.9% and 61.8%, respectively. The RSDs of the temperature and pressure of the 50 repeated experiments calculated by the LM and the MFW-LM algorithms were reduced from 2.4% to 1.1% and from 3.4% to 1.7%, respectively; that is, the measurement uncertainties of temperature and pressure calculated by the MFW-LM algorithm were dramatically reduced by 53.3% and 43.5%, respectively, compared with that calculated by the LM algorithm.

### 4.2. Reconstruction of Laser-Beam Profile

We used the MFW-LM algorithm to reconstruct a laser-beam profile from heavily distorted observations to capture more optical signal details lost in the unsaturated laser-beam profile obtained with a neutral density filter or a beam splitter in front of the image sensors.

**Figure 6:**
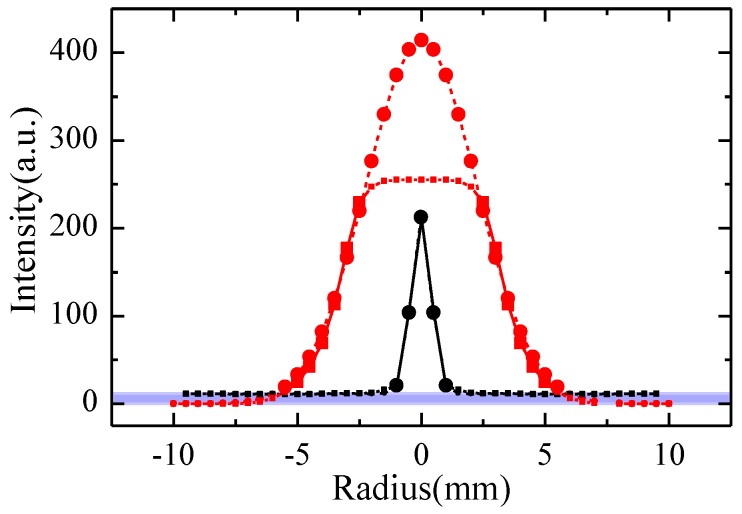
Comparison of the detail resolution of laser-beam profiles obtained by reducing laser intensity with usual means and reconstructing saturated laser-beam profiles with the MFW-LM algorithm, which are characterized by black and red curves, respectively. Data falling into the purple area have very low quality, and are represented by smaller points than those used to represent high-quality data. The solid curves represent raw signals and the dashed curves represent fitted signals.

Laser-beam profile is usually measured by image sensors to improve detection efficiency. At present, the analog-to-digital converter of an image sensor is mostly 8- or 10-bit, which means that the dynamic range (DNR) of a sensor is only 256 or 1024. In order to avoid image sensor saturation, the usual practice is to pick off a small fraction of the beam with a neutral density filter or a beam splitter in front of the image sensors, resulting in the serious loss of laser-beam profile details and the inaccurate reflection of laser-beam spatial characteristics. For example, due to the pixel resolution of the image sensor, only a few intensity points can be resolved within the dramatically changing range of the laser-beam profile, and the analysis of the laser-beam profile based on a small amount of data is considered to be unreliable. If we do not do this, even if it causes slight saturation on the laser-beam profile, the MFW-LM algorithm has been simulated and verified to accurately reconstruct the laser-beam profile, so that more intensity points of the laser-beam profile can be distinguished, giving us a chance to mine hidden information (as shown in Figure 6).

When measuring the laser-beam profile, we increased the incident-laser intensity (or the integration time) beyond the DNR but below the damage threshold of the image sensor to reflect the low intensity in details, which inevitably results in saturation and distortion. We then completed a high-precision reconstruction of the laser-beam profile by using the MFW-LM algorithm, which was proved to solve these problems.

The experimental setup was part of the research on mid-infrared laser-transmission characteristics in hollow waveguides (HWGs). The laser beam emitted from an interband cascade laser was collimated with an aspheric lens and coupled into a HWG by an off-axis parabolic mirror. An infrared camera (FLIR ThermoVision A40, Wilsonville, OR, USA) was used to measure the laser-beam profile near the waist.

**Figure 7:**
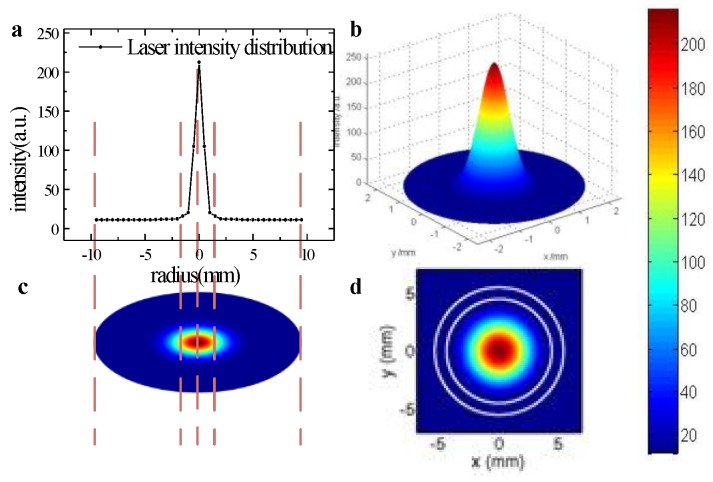
(**a**) Experimental curve of laser-beam profile; (**b**) three-dimensional laser-beam profile; (**c**) two-dimensional laser-beam profile; (**d**) HE_11_ model.

To ensure that the laser-beam profile in the experiment could be described by a Gaussian function, we obtained the intact laser-beam profile with a neutral density filter or a beam splitter in front of the infrared camera and described it with two- and three-dimensional images, as shown in Figure 7. In our numerous verification experiments, fitting degrees of the laser-beam profile and Gaussian function were all greater than 95.3%. Therefore, a Gaussian function was used to fit the laser-beam profile.

**Figure 8:**
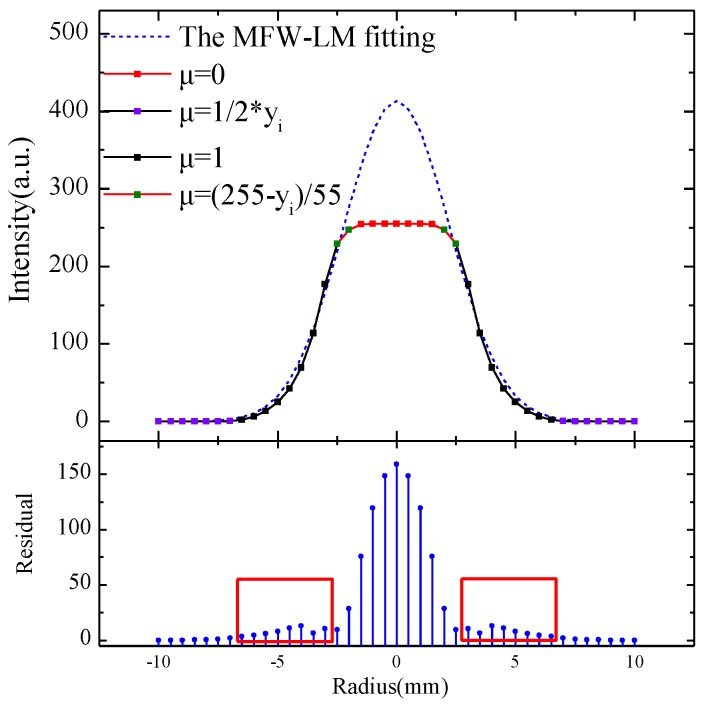
Laser-beam profile reconstructed using the MFW-LM algorithm. Data points with different colored dotted solid curves represent the fact that they were given different weights according to the corresponding membership function; dashed blue line is the fitted curve; the bottom panel shows fitting residuals and the most valuable data marked by red rectangles.

We increased the DNR of the infrared camera equivalently by reducing the attenuation of laser intensity via an attenuation device and used the proposed MFW-LM algorithm to fit the observations, as shown in Figure 8. The dotted solid curve in the upper panel of Figure 8 is the raw data and the dashed blue curve is the curve fitted by the MFW-LM algorithm. The parameters of the membership function, following Equation (1), were set as in Table 6.

The bottom panel of Figure 8 shows the fitting residuals. The data in the areas marked by the red rectangles have the highest fidelity. The relative NRMSD of the MFW-LM algorithm in the red rectangular areas was as low as 4.1% [Equation (7)].

The laser-beam profiles measured and reconstructed in the case in which the DNR of the infrared camera was increased equivalently were compared using a neutral density filter or a beam splitter in front of the image sensors for signal measuring, as shown in Figure 7. The larger points represent the data with high quality and credibility, while the smaller points indicate that the data had low quality in the purple area. From Figure 6, we note the following clarifications:

- After reconstructing the heavily distorted laser-beam profiles, the detected laser intensity at the edge of the spot was closer to 0, while the detected laser intensity in other areas was significantly increased, resulting in a significant improvement of the data’s quality.
- The number of high-quality points used to study the laser-beam profile increased from 5 (larger points on the black fitted curve) to 23 (larger points on the red fitted curve), making the detail resolution of the laser-beam profile study significantly improved by 360% and the fitting results more accurate, which can provide more extracted information for the study of the spatial characteristics of the laser beam.

As the DNR of the infrared camera increased from 256 to 415 with the proposed MFW-LM algorithm, the detail resolution of the laser-beam profiles increased by an amazing 360%. Moreover, the MFW-LW algorithm ensured that the deviation between the fitting result and the raw data in the most reliable region was as low as 4.1%. Therefore, the MFW-LM algorithm achieved high-precision measurement and high dynamic-range imaging (HDRI) to capture optical signal details.

## 5. Conclusions

In the work reported in this paper, we devised a membership function from fuzzy logic for the first time to evaluate the fidelity of optical sensing. The membership function was further used as a weighting function for the Levenberg–Marquardt algorithm. We verified the robustness and effectiveness of the proposed MFW-LM algorithm through simulation and practical sensing applications. The MFW-LM algorithm dramatically improved the NRMSD by 66.0%, more than the OE-LM algorithm for distorted optical sensing. We leveraged the great advantages of the MFW-LM algorithm in optical sensing under expected mathematical model constraints by reducing the effect of outliers and distorted signals: (1) the measurement uncertainty of temperature and pressure calculated by the molecular spectral line modeling was reduced dramatically, by 53.3% and 43.5%, respectively, compared with the LM algorithm; (2) the detailed resolution of the laser-beam profiles increased by an amazing 360% by reconstructing the laser-beam profile with the method, achieving HDRI to capture optical signal details. Therefore, the proposed method is suitable, particularly for in situ and online sensing under expected mathematical model constraints, for cases in which small datasets cannot imply a normal distribution and the deletion of any outliers and distortion would not be practical. The MFW-LM algorithm provides a useful tool for optical processing from outliers and distorted signal and has great potential for processing optical signals without depending on the quality of the measured signal.

## Figures and Tables

**Table 1:** Parameters of the Gaussian function in Equation (5).

*I*_0_	*η*	*w*	*x_c_*
−2.70 × 10^−5^	2307.40	4.45	−5.26 × 10^−18^

**Table 2:** Membership function parameters for different measured laser beams.

	*y*_min_ (a.u.)	*y*_1_ (a.u.)	*y*_2_ (a.u.)	*y*_max_ (a.u.)
**I**	16	150	150	225
**II**	3	216	216	324
**III**	45	163	163	245
**IV**	1	203	203	305

**Table 3:** The polynomial coefficients of Equation (11).

Polynomial Coefficient	0.227 < R < 0.507	0.507 < R < 2.237	2.237 < R < 7.335	7.335 < R < 62.224
*k*_1_	2285.77372	1078.16869	632.97533	417.64245
*k*_2_	−10,756.59361	−1126.15794	−129.37278	−12.094
*k*_3_	31,321.80162	908.97286	27.12434	0.4159
*k*_4_	−44,842.03108	−357.95439	−2.90883	−0.00689
*k*_5_	25,134.01199	53.92365	0.1225	4.23798 × 10^−5^

**Table 4:** Membership function parameters.

*y*_min_ (a.u.)	*y*_1_ (a.u.)	*y*_2_ (a.u.)	*y*_max_ (a.u.)
0.0	1.4	1.4	2.1

**Table 5:** Comparison of fitting results and measurement uncertainty of gas temperature and pressure from using LM and MFW-LM algorithms.

Algorithm	NRMSD (%)	Temperature			Pressure		
		Value ^1^ (K)	Value ^2^ (K)	Relative Error	Value ^1^ (atm)	Value ^2^ (atm)	Relative Error
MFW-LM	1.0	350.00	347.21	0.8%	1.18	1.16	1.4%
LM	0.9	350.00	341.02	2.6%	1.18	1.10	6.8%

^1^ Value is the truth value. ^2^ Value is the calculated value.

**Table 6:** Membership-function parameters.

*y*_min_ (a.u.)	*y*_1_ (a.u.)	*y*_2_ (a.u.)	*y*_max_ (a.u.)
0	2	200	255
